# Supplementary material for: Medical students in distress: The impact of gender, race, debt, and disability
Source: PLoS One. 2020 Dec 3;15(12):e0243250. doi: 10.1371/journal.pone.0243250 (PMC7714351; doi:10.1371/journal.pone.0243250)
Supplement: S1 Appendix — (DOCX) [file pone.0243250.s001.docx]

Medical Student Well-being Survey

**This is a national survey aimed at addressing the lack of data regarding the current state of medical student well-being. The survey is 5 pages and takes approximately 5-7 minutes to complete. All information from survey responses is completely confidential and will be analyzed in order to develop specific system-wide interventions to improve medical student well-being.**

**Section 1: Medical School & Stage**

1.01 What medical school are you currently attending?

________________________________________________________________

1.02 What stage in medical school are you currently in?

- Pre-Clinical Coursework (usually 1st Year, occasionally 2nd Year or MD/PhD) (9)
- Have begun participating in Core Clerkships (usually 3rd Year, occasionally 2nd Year or MD/PhD) (3)
- Have completed Core Clerkships (usually 4th Year, occasionally 3rd Year or MD/PhD) (4)
- Currently on a Gap Year (e.g. Research, MBA, MPH, etc.) (10)
- Other: (6) ________________________________________________

**Section 2: Medical Student Well-Being Index**

Over the last month, have you...

2.01 Felt burned out from medical school?

- Yes (1)
- No (2)

2.02 Worried that medical school is hardening you emotionally?

- Yes (1)
- No (2)

2.03 Often been bothered by feeling down, depressed, or hopeless?

- Yes (1)
- No (2)

2.04 Fallen asleep while sitting inactive in a public place?

- Yes (1)
- No (2)

2.05 Felt that all things you had to do were piling up so high that you could not overcome them?

- Yes (1)
- No (2)

2.06 Been bothered by emotional problems (such as feeling anxious, depressed, or irritable)?

- Yes (1)
- No (2)

2.07 Has your physical health interfered with your ability to do your daily work at home and/or away from home?

- Yes (1)
- No (2)

**Section 3: Demographics**

3.01 What is your current age?

- Less than or equal to 21 (1)
- 22 - 24 (5)
- 25 - 27 (2)
- 28 - 31 (3)
- Greater than or equal to 32 (4)

3.02 How do you currently describe your gender identity?

- Female (1)
- Male (2)
- Transgender (3)
- Other (4)
- I’d Prefer Not to Say (5)

3.03 How do you identify your race/ethnicity? (Select all that apply)

- Asian (1)
- Black/African (2)
- Caucasian (3)
- Hispanic/Latinx (4)
- Native American (5)
- Pacific Islander (6)
- Prefer Not to Answer (7)
- Other: (8) ________________________________________________

3.04 Do you currently identify as having a disability/chronic illness?

- Yes (1)
- No (2)
- I'd Prefer Not to Say (3)

3.05 What is your current marital status?

- Never Married (1)
- Separated (2)
- Divorced (3)
- Widowed (4)
- Married (5)

3.06 How much financial debt do you currently have?

- Between $20K and $100K (2)
- Between $100K and $300K (3)
- >$300K (4)
- I'm Not Sure (5)
- I'd Prefer Not to Say (6)

3.07 What is your intended specialty? (Note: If you have multiple or are unsure, please choose your current top choice)

- Anesthesiology (1)
- Dermatology (2)
- Emergency Medicine (3)
- Family Medicine (4)
- Internal Medicine (5)
- Internal Medicine/Pediatrics (6)
- Neurosurgery (8)
- Neurology (9)
- Obstetrics and Gynecology (10)
- Ophthalmology (27)
- Orthopaedic Surgery (11)
- Otolaryngology (12)
- Pathology (13)
- Pediatrics (14)
- Pediatric Neurology (30)
- Physical Medicine and Rehabilitation (15)
- Plastic Surgery (16)
- Psychiatry (17)
- Radiation Oncology (18)
- Radiology-Diagnostic (19)
- Radiology-Interventional (29)
- Surgery (20)
- Thoracic Surgery (21)
- Urology (26)
- Vascular Surgery (23)
- Other: (24) ________________________________________________

3.08 How confident are you in your choice of intended specialty?

- 100% (1)
- 75% (2)
- 50% (3)
- 25% (4)
- 0% (5)

**Section 4: Stressors & Well-Being Experience**

4.01 How often do you participate in the following activities in an ideal week vs a realistic week?

|  | Ideally | | | Realistically | | |
| --- | --- | --- | --- | --- | --- | --- |
|  | ≥ 5 days (1) | 2-4 days (2) | ≤ 1 day (3) | ≥ 5 days (1) | 2-4 days (2) | ≤ 1 day (3) |
| Eat healthy (1) |  |  |  |  |  |  |
| Exercise (2) |  |  |  |  |  |  |
| Mindfulness practice (3) |  |  |  |  |  |  |
| Hobby (i.e. Read a book, Create art) (4) |  |  |  |  |  |  |
| Connect with a friend/loved one (5) |  |  |  |  |  |  |

4.02 How do the following items affect your stress level?

|  | Significant Decrease in Stress (1) | Mild Decrease in Stress (2) | No Change in Stress or N/A (3) | Mild Increase in Stress (4) | Significant Increase in Stress (5) |
| --- | --- | --- | --- | --- | --- |
| Grades/Evaluations (12) |  |  |  |  |  |
| National Board Exams (13) |  |  |  |  |  |
| Isolation from friends/family (1) |  |  |  |  |  |
| Gender or Gender Identity (9) |  |  |  |  |  |
| Ethnicity or Ethnic Identity (10) |  |  |  |  |  |
| Financial debt (2) |  |  |  |  |  |
| Lack of control over schedule (7) |  |  |  |  |  |
| Specialty of choice (4) |  |  |  |  |  |
| Concern about workload in residency (5) |  |  |  |  |  |
| Uncertainty about future (8) |  |  |  |  |  |
| Disability/Chronic illness status (6) |  |  |  |  |  |
| Caregiving responsibilities (3) |  |  |  |  |  |

4.03 Which of the following activities do you use to cope with difficult situations (or a difficult day on clinical rotation)? (Select all that apply)

- Talk to a friend/loved one (1)
- Consume alcohol or marijuana (2)
- Consume other drugs (7)
- Exercise (3)
- Watch TV/movie (4)
- Lie down (5)
- Listen to music (6)
- Mindfulness practice (8)
- Meet with a counselor (9)
- Other: (10) ________________________________________________

4.04 How do you feel your well-being has changed in the following domains since beginning medical school?

|  | Significant Decrease (1) | Mild Decrease (2) | No Change (3) | Mild Increase (4) | Significant Increase (5) |
| --- | --- | --- | --- | --- | --- |
| PHYSICAL WELL-BEING (1) |  |  |  |  |  |
| SOCIAL WELL-BEING (2) |  |  |  |  |  |
| EMOTIONAL WELL-BEING (3) |  |  |  |  |  |

4.05 Please mark the statement you most identify with:

- I have considered taking a Leave of Absence from school for my personal well-being (1)
- I have taken a Leave of Absence from school for my personal well-being (2)
- I have never considered taking a Leave of Absence from school for my personal well-being (3)

**Section 5: Medical School Characteristics**

5.01 What is your school's grading system during **Pre-Clinical Courses**?

- Pass/Fail ONLY (1)
- Pass/Fail + Recognitions for Honors/High Pass (2)
- Letter Grades (A, B, C, etc.) (3)
- Other: (4) ________________________________________________

5.02 How supportive do you feel your school's faculty are in terms of well-being?

- Not Supportive at All (1)
- Somewhat Supportive (2)
- Strongly Supportive (3)

5.03 At your institution, which of the following well-being resources are available? (Select all that apply)

- Mental Health & Counseling Services (1)
- Peer Mentorship (2)
- Self-Care Education (4)
- Mindfulness/Meditation Classes (5)
- Community Building Events (3)
- Other: (6) ________________________________________________

Carry Forward Selected Choices - Entered Text from "At your institution, which of the following well-being resources are available? (Select all that apply)"

5.04 At your institution, which of the following well-being resources have you utilized? (Select all that apply)

- Mental Health & Counseling Services (1)
- Peer Mentorship (2)
- Self-Care Education (3)
- Mindfulness/Meditation Classes (4)
- Community Building Events (5)
- Other: (6)

5.05 What well-being resource(s), if offered at your school, do you feel would be most useful?

________________________________________________________________

5.06 If you have any further comments to share, please write them below:

_____________________________________________________________

**Section 6: Interview Recruitment**

6.01 Are you willing to be contacted for a short interview on your experience with medical student well-being?

- Yes (4)
- No (5)

Display This Question:

If Are you willing to be contacted for a short interview on your experience with medical student wel... = Yes

6.02 Please enter your school email address below:

________________________________________________________________
