# Supplementary material for: Medical students in distress: The impact of gender, race, debt, and disability
Source: PLoS One. 2020 Dec 3;15(12):e0243250. doi: 10.1371/journal.pone.0243250 (PMC7714351; doi:10.1371/journal.pone.0243250)
Supplement: Attachment — Submitted filename: PLOS_Response to Reviewers_v2.docx [file pone.0243250.s003.docx]

Response to the Reviewer

We thank you for your time and insightful critiques about our manuscript. We appreciate the overall positive feedback and we hope to strengthen our manuscript by addressing your concerns.

1. **Survey validation.** In developing our survey instruments, we scoured the literature for previously validated questions that contribute to medical student wellbeing and burnout. Unfortunately, no single survey questionnaire contained what we were searching for and many wellbeing surveys were designed for either older populations, where chronic health conditions and dependence are more prevalent, or corporations, where productivity and distractions are of higher concern. As such, we opted to create a novel instrument centered primarily around the MS-WBI. Additional questions added to our survey were designed under the guidance of previously validated surveys, utilizing Likert scales and topics previously known to contribute to burnout and stress. These foundational surveys have been included as references in our manuscript in addition to a clarifying statement.
2. **Survey piloting.** We appreciate the reviewer addressing our oversight in not describing the piloting of the survey. The survey was piloted by 10 medical students from various institutions to ensure the survey was of adequate length, easy to comprehend and comprehensive. After an initial round of feedback regarding the survey, we made several edits and then underwent a second round of piloting, at which point there was universal acceptance of the survey for all participants. A description of this process has been included in the manuscript. A copy of the survey has been provided as supplemental material for the manuscript.
3. **Medical student liaisons.** We have added the total number of medical student liaisons to our manuscript.
4. **Rationale for disability in the regression model.** We included disability in our regression model for severe distress as disability amongst medical students has risen substantially over the last decade and the effects of disability on wellbeing was previously unknown. Our study demonstrates that identifying as having a disability is a risk factors for having severe distress. We believe that this finding contributes significantly to the literature and we hope that publishing our results will better inform medical schools and help design interventions to mitigate this risk. We have included additional information about disability amongst medical students in our introduction.
