# Supplementary material for: Medical students in distress: The impact of gender, race, debt, and disability
Source: PLoS One. 2020 Dec 3;15(12):e0243250. doi: 10.1371/journal.pone.0243250 (PMC7714351; doi:10.1371/journal.pone.0243250)
Supplement: Attachment — Submitted filename: PLOS_Response to Reviewers_Round2.docx [file pone.0243250.s004.docx]

Response to the Reviewer

We thank you for your time and insightful critiques about our manuscript. We appreciate the overall positive feedback and we hope to strengthen our manuscript by addressing your concerns.

1. **Abstract Correction.** We appreciate the reviewer addressing our oversight in not including disability in the conclusion of the abstract. We have added disability status as a novel risk factor to the abstract conclusion.
2. **Updated Citations.** We have included updated citations on student depression/suicidal ideation as indicated.
3. **Disability/Chronic Illness Status.** We have added the fact that one of our novel questions was asking about disability/chronic illness status. We also included that a higher number of students self-identified as having a disability than has been found in prior literature possibly due to student discomfort.
